# Becoming a matter of veterinary concern

**DOI:** 10.3389/fvets.2024.1355996

**Published:** 2024-05-30

**Authors:** Rebecca Smith, Gina Pinchbeck, Catherine McGowan, Joanne Ireland, Elizabeth Perkins

**Affiliations:** ^1^Department of Equine Clinical Science, Faculty of Health & Life Sciences, Institute of Infection, Veterinary and Ecological Sciences, University of Liverpool, Liverpool, United Kingdom; ^2^Department of Livestock and One Health, Faculty of Health & Life Sciences, Institute of Infection, Veterinary and Ecological Sciences, University of Liverpool, Liverpool, United Kingdom; ^3^Department of Primary Care and Mental Health, Faculty of Health & Life Sciences, Institute of Population Health, University of Liverpool, Liverpool, United Kingdom

**Keywords:** ageing, health care, horse–human relationships, sociology, veterinary medicine

## Abstract

Horses in Great Britain are living into increasingly older age and are often regarded as friends or family members by their owner. The horse is reliant on their owner to meet their needs and this paper discusses how horse owners frame an issue that becomes a matter of veterinary concern within the context of the older horse. Qualitative methods were used to explore the experiences of owners and veterinarians. Data were collected and analysed using a grounded theory approach during the period 2019–2022. Analysis identified that owners undertook an ongoing and iterative process of assessment, monitoring and decision making in relation to the animal and any changes they observed. Matters that became a veterinary concern required the owner to formulate the issue as something that fell within the knowledge domain of the veterinarian. Veterinarians had a medicalised view of older horse health and their perspectives on socially acceptable care were shaped by their understanding of species-specific needs, and whether owners were providing appropriately for those needs. The formulation of a matter of veterinary concern was itself shaped by an owner’s experiential knowledge of both veterinary matters and their horse. The extent to which owners felt like they and their individual horse mattered during interactions with veterinarians affected whether they adopted veterinary advice and the nature of future veterinary employment. Findings demonstrate how matters of health, disease, and the role of professionalised forms of medical knowledge, are not static but constantly changing and interacting over time. An issue that became a matter of veterinary concern was contextual, and rooted in individual relationships. The significance of veterinarian-owner interactions in shaping future consumption of veterinary health care may be underestimated.

## 1 Introduction

We live in a world of interwoven multispecies relationships (1, 2). Issues of everyday life are in a constant state of “becoming” within the networks of social, material, and political structures in which they arise (3) and animals play an important role in the creation of health knowledges (4). While humans and animals live together in society and many are viewed as family members (5), society does not take responsibility for animals which are seen as privately owned. The state steps in where the actions of owned animals disrupt everyday life, e.g., dog fouling, dog bites, zoonotic diseases, but in the main, the animal remains the sole responsibility of their owner.

From the perspective of the veterinary profession, veterinarians play a central role in animal care and preside over the body of knowledge and expertise associated with animal health and welfare (6). Society grants veterinarians legal powers to prescribe, treat and conduct certain technical procedures on the basis of their education and training. However, epidemiological research has reported that there is a reduced uptake of “routine” veterinary services (namely for the provision of vaccinations) by owners of older horses as their horse ages and upon a horse’s retirement (7, 8). This change in the use of veterinary services was used as the point of departure in this study. This paper explores the relationships between an older horse, their owner, and their veterinarian, to understand how networks are drawn upon in decision making regarding the consumption of health care services and why an issue becomes a matter of veterinary concern.

Many people (hereafter, owners) are caring for their horse into old age (9, 10). The equine life course is often thought of in similar ways to that of humans, with socially constructed phases of education, work and retirement. Studies indicate that owners of older horses report changes such as increasing grey hair, stiff joints or lack of joint flexibility, loss of muscle tone and deepening of supraorbital hollows, and often attribute these to signs of ageing (8, 11). The veterinary-reported prevalence of chronic disease amongst the older horse population is considered to be high (12). However, literature reports differences between owner-reported and veterinary-identified signs of disease (13, 14), suggesting that there may be divergent views on the meaning of disease and its amenability to treatment. These observations, along with the reduced uptake of routine veterinary health care services by owners as their horse ages, suggest that lay constructs of disease and perceptions of the role of the veterinarians in older horse care, do not necessarily align with professional notions.

Veterinarians are dependent upon animal owners for their employment. For animal owners to employ the services of a veterinarian they have to frame an animal’s need as a matter of veterinary concern. The skills and competencies of the veterinary profession reach into many areas of an animal’s life, including preventive health practices and areas that may be deemed relevant to the good practice of life (15). Whether or not an animal owner chooses to consult a veterinarian and follow their advice depends upon a whole host of complex factors.

Veterinary services are reported to be just one of many sources available to owners to assist in the management of their horse’s health (16, 17). An owner’s networks of advice and support may include; peers, livery (horse housing) staff, online communities, friends and health care providers such as physiotherapists, and veterinarians (18). Exactly where the veterinarian fits into the care of the older horse varies enormously between owners, and in relation to the horse in question. When asked, owners report valuing the veterinarian’s opinion in relation to expensive or long-term veterinary care and euthanasia decision making (10, 19). However, the timing and nature of veterinary involvement varies. A study of Australian horse owners reported that veterinarians were consulted for serious issues or as a last resort, and there was little mention of their role in preventive health care (20). A study examining horse owner approaches to responding to equine colic found that three (not mutually exclusive) strategies were adopted: “wait and see”, “lay treatment” or “veterinary assistance” (21). Therefore, veterinary involvement is likely to take place alongside other management strategies.

Where a veterinarian is involved in an animal’s care their decision making is reportedly influenced by the nature of the animal carer’s wishes alongside the animal’s health (22, 23). In the context of the older horse, there appears to be a focus on identifying diseases that have become associated with old age. Literature on older horse health and husbandry uses language such as aged, geriatric or senior, reflecting an association with decline (10, 24, 25). A small survey of Austrian veterinarians indicated that advanced horse age eased their management of euthanasia decisions (26). Whilst differences in owners’ approaches to involving a veterinarian are clear, the role of the veterinarian in shaping these approaches, has not previously been explored.

The approaches of, and interactions with, medical professionals are known to be linked to health outcomes. In the human health care context, cultural meanings regarding matters of health and disease are known to differ between groups, affecting the reported prevalence of a particular condition. Where a disease is contested, this may for example result in users of health care services being resistant to being labelled as having that disease, or where patient experiences do not fit within a medical construct, left being unable to access treatment (27). The concept of mattering—feeling significant, valued and heard by other people—and its significance as a predictor of outcomes such as academic performance, academic stress, life satisfaction, and happiness has been described (28). Therefore, this paper is concerned with the interactions of different social groups (horse owners and veterinarians) and their respective health knowledge(s) and the extent to which this affects owners’ uptake of, and future engagement with, veterinary health care services.

This exploratory research sought to understand how owners of older horses made decisions regarding their horse’s management and health care provision. This paper draws on empirical data to discuss how owners’ experiences impact on their decision making and how issues with their horse are constructed as a matter of veterinary concern.

## 2 Methods

This paper draws on data collected as part of a wider study that examined how horse owners and veterinarians make decisions regarding care of the older horse. The research was reviewed and approved by the University of Liverpool’s Veterinary Research Ethics Committee (reference VREC901).

This research was underpinned by a social constructionist epistemology in order to understand the way in which people construct their realities, the meaning they take from them and how this shapes their decision making. A symbolic interactionist theoretical perspective was used to enable the exploration of how people’s attitudes and beliefs changed with time and context. This study adopted a constructivist grounded theory methodological approach as described by Charmaz (29) in order to generate theory from data (30).

### 2.1 Data collection

Multiple sources of qualitative data were purposively sampled from participants located in different regions across Great Britain. The collection of data included: 12 online open-access discussion forum threads containing 326 comments (open-access, based in Great Britain, active during 2016–2020 see (18) for further details); 25 semi-structured interviews with owners of older horses, nine semi-structured interviews with respective veterinarians and 13 sets of veterinary clinical records pertaining to the interviewed owner’s horse(s) covering the previous 2 years (collected during the period 2020–2022). Horse owners were recruited for interviews using an online advertisement and veterinarians were recruited based upon their involvement in the care of the owner’s horse. All participants responding to the advertisement, as well as the veterinary practices/veterinarians contacted directly, were provided with a study information sheet. One participating owner and one veterinarian were known to Rebecca Smith (RS) beforehand, while all other interviewees had no prior relationship. There were a few owners who had no veterinarian to nominate, and one veterinarian declined to be contacted for interview when the participating owner had mentioned the study to them. In some instances, the veterinary practice submitted the clinical records pertaining to the interviewed owner’s horse but the veterinarian was not interviewed, or vice versa. Reasons for being unable to obtain clinical records or interview the nominated veterinarian included a lack of response or reported time constraints.

Most interviews were held online or by telephone due to restrictions related to the COVID-19 pandemic. Following introductions and opportunity for participants to ask questions about the study, RS obtained informed consent for participation (see Supplementary Data 1). All interviews were audio-recorded. Interviews followed a semi-structured approach with an interview topic guide used to prompt discussion of relevant topics. Follow-up questions varied depending on responses during each interview (see Supplementary Data 2). Interviews with owners were generally around 60–90 min duration, whilst interviews with veterinarians were mostly shorter, of around 45–60 min. As part of the wider study, a number of retirement livery premises were visited (once travel restrictions lifted), and one veterinarian was recruited via this route. Fieldnotes including reflections and initial impressions were written by RS following the interviews and fieldwork.

### 2.2 Data analysis

The analysis of data took place alongside its collection using a constructivist grounded theory approach (29). RS was primarily responsible for collecting and analysing all data. Data were anonymised before being inductively coded—fractured down to words, phrases or lines—and conceptual labels or “codes” applied. These were grouped in conceptual categories and their relationships interrogated to create conceptual models. Coding and theory development was discussed in-depth with Elizabeth Perkins (EP) and frequent discussions took place throughout the project with the whole research team. Constant questioning and comparison of data enabled analysis to move in new theoretical directions and drove theoretical sampling. This enabled greater detail of decision-making processes and the properties of categories to be developed which produced a dense theory that was grounded in people’s experiences (29–31). This paper presents a substantive theory about decision making in relation to veterinary involvement in older horse care.

### 2.3 Reflexivity

RS engaged in an ongoing process of reflexivity as data collection and analysis evolved. RS, a female veterinarian with experience of caring for (but not owning) horses was, at the time, a PhD scholar trained in social research methods. This role as a relative ‘outsider’—being a small animal, rather than equine, veterinarian—enabled the questioning of colloquial language and worldviews during the initial coding process. These experiences and understanding also assisted in building rapport with interview participants. The research team also included EP, a social scientist with experience in health and social policy research and herself a horse owner, as well as three veterinarians with expertise in epidemiology and equine medicine who had previous experience of working in multidisciplinary teams on qualitative research projects.

## 3 Results

Analysis identified that, in their life with their horse, owners undertook an ongoing and iterative process of recognising and responding to change on a daily basis. The role of the veterinarian, and the way in which matters were understood to be of veterinary concern, was situated within this context and shaped by past experiences with veterinarians. This paper presents four interrelated themes through which the factors that shape, and the consequences of, decision making within these networks of relationships are discussed. Firstly, the process of recognising and responding to change will be described. In part two, the ways in which issues were raised to those perceived to require veterinary attention are discussed. The third section presents findings on how (sometimes differing) perspectives were generated regarding what made an issue a matter of veterinary concern. In the final section, the consequences of veterinarian-owner interactions for horse health, and for owners’ views of the role of the veterinarian, are presented.

### 3.1 Recognising and responding to change

Over time through their interactions with their horse, owners developed knowledge about each horse as an individual. Many owners had established daily routines of care, creating a normative understanding of their horse. Deviations from this norm raised questions for the owner about their management of the horse and the reasons for this change:

“We controlled the laminitis [painful condition affecting the tissues of the horse’s foot] fine and then it just felt like, I don’t know, I wouldn’t say he had laminitis, he just started to look a little bit footy and you think, he had the same routine and suddenly the routine wasn’t working as well.” (Sarah, owner).

Owners processed information about a horse’s changing condition in their everyday context and made early attempts to attribute meaning to the changes they observed. For example, one owner Jill talked about noticing a change in her horse’s gait. She interpreted the meaning of this in the context of the environment and her knowledge of her horse’s physical and mental characteristics. This understanding was also influenced by how the horse changed over time:

“Umm, so it was slightly pitted from the winter poaching…just slightly at one end and uh, I saw him walking and he was pottery…but he was pottery all round, you know and I kept thinking is it the ground, cos he does he is a bit of a ponse you know for um sort of delicate feet you know, uh doesn’t like walking on gravel things like that you know. um and uh I sort of, but within a couple of days the rain had come back, ground had gone a little bit softer so I thought maybe it was him just being like that.” (Jill, owner).

As Jill demonstrates in her quote above, owners look to common sense explanations first before settling on a course of action. Within a dynamic model, care could be adapted to fit everyday changes that took place. Subtle changes however, were sometimes reported to be difficult to recognise, especially in the context of caring for a group of horses.

Owners adopted strategies in order to resolve issues and these differed depending upon their experience and the perceived severity and urgency of the issue. Owners commonly talked about increasing their monitoring of the horse, or a particular issue of concern, during the problem-solving process. Increased monitoring allowed an owner to establish whether an issue was of concern. This ‘watch and wait’ could be the only strategy adopted or could take place alongside management changes:

“So, every 2 hours I would go up and check on her, because I was terrified of her going down in the stable and not getting up.” (Emma, owner).

The process of recognising and responding took place continually over time, and therefore, problem-construction was an iterative process:

“Well when he first started itching I thought he’d got lice. Then I thought, “Oh no, it’s sweet itch [allergic reaction to insect bites]” Then it went on all through winter, and I thought, “No, it can’t be sweet itch.” I was just sort of trying everything really.” (Lorna, owner).

Knowledge of a particular horse and through extension, horses in general, was developed through experience and reflection on past experience. One participant described how she had previously managed recurrent colic in her mare. The recurrent nature of colic, by contrast with another horse and with hindsight, led the owner to label the horse as ‘a colicky horse’:

“I guess I reacted if she was ill. She was quite a colicky horse. She suffered … I wouldn’t have said that, at the time, but having Magic now who is not colicky at all, I can look back and think, ‘Yes’.” (Susanne, owner).

Although owners could recognise a change, social influences shaped whether a change was considered to be problematic or not. For example, the livery yard environment (where multiple horse owners share use of a premises) could, in some circumstances, be helpful in attributing a cause of the horse’s issues:

“Well, he dropped quite a lot of weight and muscle quite quickly and the yard I was on, at the time, one of girls said, “Have you thought about getting him looked into for Cushing’s [Endocrine disorder Pituitary Pars Intermedia Dysfunction (PPID)]?” But I didn’t know a great deal about it there were things I’d seen about it like maybe get a curly coat and stuff like that, so I was like, “Hmm, I’ve not.” (Mary, owner).

Monitoring change over time was an important feature of care in this study. In instances where horses developed chronic conditions, owners developed individual ways of monitoring their horse, and in turn, of recognising change. One owner valued riding her horse in order to pick up changes:

“Because I can’t tell from the ground completely. I’ve always been able to pick up his lameness very, very quickly when I’m on him. You can’t see it when he’s walking around the field.” (Patricia, owner).

The environment, facilities and resources available to an owner shaped how management changes were made. For one owner who cared for multiple ponies, finances were a consideration and dental assessments were not part of her ‘routine’ care provision for her horses. In response to her perception of a dental issue she adopted a lay management strategy, namely, the adjustment of her pony’s feed. The owner spoke about the fact that her pony had responded well to this, and for her, this meant that the pony did not require professional dental assessment unless anything else changed:

“With Jimmy, it was coming up to autumn, into winter last year that his condition dropped quite drastically, quite quickly. It was to do with his teeth. Now, I haven’t had his teeth checked out, but he now gets a feed supplement to maintain his condition. He’s on Veteran Vitality, a veteran mix and Chaff as well. He’s getting a decent feed … He’ll probably get his teeth checked out the next time that his injections are due unless he changes. But he’s doing well.” (Leah, owner).

In instances where solutions to problems were unclear, owners sought advice from those deemed to have relevant knowledge. Depending on the owner’s individual understanding of what type of issue warranted veterinary advice meant their advice-seeking behaviours might be directed differently. This could be through independent research or study, or through speaking to friends or professionals. For some owners, experiential knowledge was valued and sought from peers via online equestrian communities:

“My elderly mare has started having seizures. I've been told that the likely cause is a tumour. Vet has been talking about possible treatment, but I feel very strongly that I don’t want to put her through lots of invasive tests and pump her full of drugs … Anyone been in a similar position?” (forum user).

Sources of advice were adopted differently depending on availability and the perceived relevance of knowledge for that particular issue.

### 3.2 Becoming a matter of veterinary concern

The way in which veterinarians were employed partly depended upon an owner’s understanding of their horse’s need for a health care measure; for example, vaccination or dental treatment. While some services could only be accessed from a veterinarian, dental care could also be performed by other professionals such as equine dental technicians. Therefore, decisions made by the owner determined who was then able to provide a particular service for the horse. In addition to such health care services, veterinary advice could be sought for problems perceived to be specifically veterinary-related, or if the horse was ‘just not right’ and the owner believed that veterinary knowledge would assist in resolving the issue. However, what made an issue a matter of veterinary concern was not straightforward and varied between individuals. Perceptions of the role of the veterinarian were influenced by past interactions and factors including the veterinarian’s communication style, technical skills, medical knowledge and their interaction with the horse. While in theory, veterinary visits were opportunities for evaluation and planning for a horse’s long-term care, some owners reported that veterinarians did not use consultations as an opportunity to find out about the horse:

“They didn’t ask me any questions at all about what they did, what they didn’t do. And I found that really odd and I said to Nathan, “I don’t think I’m going to like him as a vet.” They’ve no interest whatsoever in anything about the horse, they just came down, gave him an injection and went. (Emma, owner).

Some owners saw regular (annual tetanus or biennial influenza) vaccination as an essential part of care which reflected their ongoing commitment to the horse into older age. Others believed that regular vaccination was unnecessary because previous vaccinations conferred life-long immunity for their aged horse. Alongside a reduction in perceived risk—often related to reduced or more localised activities with the horse, or few horses entering or leaving a premises—some owners stopped influenza vaccination, or vaccinations entirely, as the horse aged and their lifestyle changed. The perceived necessity of involving a veterinarian for such measures was also related to an owner’s ideas about what expertise, and type of service, their veterinarian could provide.

Owners knew in what instances they would go to a veterinarian for issues that arose; however, this had individual meaning and could change over time. During the process of ‘watch and wait’ owners used individual and specific ways of actively monitoring their horse’s health. This knowledge was then used to know when to involve their veterinarian:

“We’ve, kind of, managed to keep it in check since then, but it was the scabby legs that we noticed. And she does still have it. It’s not as bad as it used to be, and it doesn’t seem to bother her, but when it gets a bit worse, we usually get her tested again, because you quite often find that her levels have gone up again.” (Savannah, owner).

Owners used their past experience as a basis for managing certain issues and this knowledge could form the basis of actions that replaced ‘watch and wait’. Owners were willing to substitute this experience for a veterinary consultation, particularly where there was a similarity between the past and a current presenting issue:

“I think it just goes on experience really, like you sort of, you know with certain things that the advice you’re probably going to get is box rest and bute [non-steroidal anti-inflammatory drug], so if you have them to hand I normally would give them a try at first. Unless it’s something dramatic like when he couldn’t put his foot down and then I’d be straight on the phone to the vet.” (Mary, owner).

Where unable to resolve issues by themselves, owners sought veterinary services in order to access medical knowledge, diagnostic equipment, technical procedures or prescription medication. Where owners had an established relationship with their veterinarian or practice, they were sometimes offered the option of discussing an issue with their veterinarian by phone or using a messaging service. Uncertainty by an owner about whether an issue was something that constituted a matter of veterinary concern could prompt enquiries before a visit was booked. For instance, if a horse was ‘just not right’ and the owner was unsure of what was causing the problem, advice could be sought prior to a consultation:

“He started just standing in the stable”. And, again, he wasn’t himself. I would be saying, “Are you okay? Are you okay? And he wasn’t, you know. So, I said to Becky [veterinarian], “Look, he is not right. I am not sure what is not right.”” (Susan, owner).

Where veterinarians became involved in a horse’s care their approach hinged on the identification of problems and finding solutions to those problems. Therefore, the way in which the owner presented the horse, and the veterinarian and owner interacted, shaped the nature of decision making that took place. Issues that constituted a matter of veterinary concern could differ between owners and veterinarians. Veterinarians felt that some owners found it difficult to identify change as a problem and to seek timely advice. Varying levels of horse-related knowledge and experience were perceived by veterinarians to impact on how owners assigned meaning to a change, which was perceived to delay advice-seeking:

“He called us out initially because he thought that the horse wasn’t moving very well and had slowed down when she was out on hacks. He is a very novice owner, which I’m sure he would admit himself. So, his complaint was that he was hacking the horse and she slowed down. I went out to see her and obviously she presented with actually quite significant hindlimb lameness which was very positive to flexion.” (Laura, veterinarian).

An owner’s understanding of a veterinary-related issue informed the course of the consultation. In some cases, owners expressed their concerns about a horse’s health in terms of a diagnosis which made the veterinarian’s first course of action more straightforward:

“Sometimes clients call us, “I think my horse might be Cushingoid. Please can you come and test them.”” (Laura, veterinarian).

The interplay between owner and veterinarian meant that the meaning of a veterinary-related problem was co-constructed during the consultation. Once within the purview of the veterinarian, the issue of concern could be reconstructed. This could present opportunities for discussion around aspects of a horse’s management, such as ridden exercise, that were relevant to the horse’s health:

“Because she was on Danilon [non-steroidal anti-inflammatory drug], regular Danilon, obviously he had to prescribe that and so he would get to see her a couple of times a year. Then we’d just talk about general management, really… Even though she was quite stiff, she used to love hacking out, and she’d still want to try and gallop off if she could, so it was Matt that said, “Actually, Isla, I think you need to retire her now,” because she was getting quite … She used to stumble sometimes, and she might be extra stiff after a ride, so he would advise me on managing her arthritis anyway, and then just generally managing her condition.” (Isla, owner).

Where time permitted, veterinarians appreciated questions from owners, believing that it reflected the owner’s motivation to look after their horse and that they valued veterinary advice. Veterinarians recognised that discussions with owners opened the possibility of exploring wider issues which might be relevant for the owner.

The time frames in which owners sought advice from veterinarians depended upon; their understanding of the problem and its urgency, response to lay management strategies, relationships with veterinarians and consultation fees, e.g., the consideration of increased fees for out of hours emergency consultations. The importance of each owner’s construction of a matter of veterinary concern had implications for the presentation to a veterinarian, and as later discussed, this presentation shaped decision making by the veterinarian. However, owners did not always restrict themselves to registering their animal with one practice. A few owners spoke about using different veterinary practices for different services—namely differentiating between vaccinations and problem-based consultations. Issues of concern regarding different aspects of the animal’s health care could result in owners directing that horse to one practice or another. Therefore, veterinary knowledge of the horse was not necessarily confined to one practice and the owner was central to the co-ordination of care.

### 3.3 Perspectives on what made an issue a matter of veterinary concern

Veterinarians’ judgements about the nature and timing of their involvement was based on their medically-informed notions of health and their ideas about acceptable ways to manage a horse in old age. These related to their understandings of how the body functions and their knowledge of species-specific needs. While owners drew on a much wider knowledge base including their own experiences with the horse over time. Apparent ‘delays’ in advice-seeking could be emotionally upsetting for veterinarians, particularly when horses were presented to them in significant pain:

“The other sad thing, I think, is they get—not on purpose—neglected more because they’re often just retired, so in a paddock, and you don’t see them walking on concrete, so they miss the early onset laminitis. And they think, “Oh, it’s losing weight because it’s old”, when it isn’t. It’s because it’s got something going on with it.” (Annelise, veterinarian).

The extent to which veterinarians sought to involve the owner in decision making during the consultation was informed by their assessment of the horse’s condition and judgements about owner knowledge—as reflected in their advice-seeking behaviours. A veterinarian’s ideas about what constituted a concern regarding the horse’s wellbeing, and a possible way to resolve it, had to be negotiated in the context of what might be possible for the owner. Veterinarians talked about many possible considerations when finding solutions to problems identified, such as the horse’s personality, owner finances, family commitments, housing environment, access to facilities, as well as the owner-horse relationship. These factors were seen as elements that necessitated the ‘negotiation’ of a form of appropriate care, which inevitably required a deviation from the veterinary ‘ideal’:

“I think basically from my point of view we would say what’s gold standard in terms of having them in and the box rest required and what they could eat, and we would probably then compromise on something that was not awful but not great. So, it was, yes, cornering off sections of paddock and having smaller paddocks, especially when they got, I think it was Gem that used to get abscesses, when she had abscesses and stuff, they couldn’t be out in the field, but we managed it with small pens and stuff. So, yes, explained gold standard and then compromised on something that was acceptable but not gold standard.” (Frank, veterinarian).

Veterinarians reported using strategies to shift an owner’s thinking into the need for extra health care interventions. In the following example this involved the suggestion of a joint supplement:

“So, I try and start them off on the Devil’s claw, Boswellia-type things first to introduce them to the idea that they need some help. Then, as soon as they see there’s an improvement and they see the horse looks happy, they soon come around quite often.” (Annelise, veterinarian).

Different perspectives on what constituted a problem, or an appropriate solution, became more salient for veterinarians when they were presented with a horse experiencing worsening chronic problems, or where additional acute problems necessitated emergency consultations. These were cases where horse welfare was a more significant and acute concern: for example, colic requiring surgery, an inability to stand, or chronic worsening issues that were significantly impacting on quality of life such as long-term musculoskeletal pain. For veterinarians, these could prompt consideration of concerns such as unrelenting pain, suffering and end-of-life. In such contexts, veterinarians felt a moral obligation to get owners to comply with their advice. However, solutions could be challenging to navigate with owners if they were not on the same page:

“And all of a sudden you’re here on a Saturday night saying, “Your horse has some sort of bad intestinal lesion and it needs to be put to sleep.” I think in those cases it’s challenging because you’ve got to get the owner from, “My horse was healthy two hours ago,” to a, “It’s essentially going to die without anything else.” So, the owner-Yes, it’s trying to explain to the owners that that is the case.” (Paul, veterinarian).

Veterinarians could feel powerless in certain situations particularly if they felt that the horse was suffering. Similarly, owners could feel powerless if they perceived that the veterinarian had not achieved an understanding of their horse as an individual. This could influence the way in which an owner received veterinary advice:

“I was frightened that I would come under intense pressure to euthanise her, and I wasn’t willing to do that. If I felt they understood my horse and had given me that advice I might have accepted it, but they didn’t understand my horse.” (Steven, owner).

The involvement of a veterinarian reflected that the owner understood the issue with their horse to necessitate veterinary expertise. However, the nature of the issue and what types of decisions it required them to make were not necessarily the same as the attending veterinarian. The way in which owners received and acted upon advice was influenced by how veterinary knowledge was situated alongside their own knowledge and beliefs, and whether successful communication had taken place:

“I didn’t really respond to it. I thought, “I understand why you’re saying that. You’re saying that because you think that’s how he’s going to get exercised.” I don’t think I responded to it. I thought, “It’s a good thing because she thinks he’s well enough to be ridden so I’ll take that as a sign that she thinks he’s well enough to be ridden but I won’t be doing it.” From memory, she doesn’t really say it now. I think she realises I’m not going to be doing it.” (Hazel, owner).

Even within longstanding veterinarian-owner relationships owners reported that there were gaps in the veterinarian’s knowledge of how a horse was actually managed on a day-to-day basis by the owner.

### 3.4 Consequences of veterinarian–owner interactions for horse health

Veterinarians could provide a source of support and reassurance for owners that socially acceptable care of the horse was being undertaken: for example, if this was questioned by other owners, professionals or the general public. However, where unfamiliar veterinarians attended, this could sometimes challenge the construction of an appropriate form of old age care. Although there was a common desire to act in the horse’s best interests, veterinarians and owners did not base their actions and decisions on the same type of knowledge:

“And then, Christine [veterinarian] left and then we got a succession of young vets. Which, technically, they were right, but the amount of painkillers she was on was at too high a dose. Which, technically, they were right, but it was keeping the pony comfortable. And, we did reduce the amount of painkillers, and, so from then, she was back into pain and she didn't live very long.” (Julie, owner).

When doing what they considered was best for their horse, owners could choose not to follow, otherwise adapt advice to their own context or seek further advice from other sources. In this study, owners interpreted the veterinarian’s advice according to their own knowledge even when they were making decisions in the context of veterinary medicine:

“Once I got him on one tablet a day then we stayed on that until recently, about three months ago, and now he’s on half a tablet one day and a full tablet the next day. So, I just alternate it. James said to drop it down to half a tablet each day, but I was a bit dubious about doing that because of the ACTH [Adrenocorticotropic hormone] levels going high at this time of year anyway. I just thought it was a bit too risky to drop it that much, just in case he got laminitis again.” (Patricia, owner).

The veterinarian’s interactions with both horse and owner influenced an owner’s preferences over which veterinarian attended. A sense of mattering for both veterinarian and owner was reflected in owners requesting a particular veterinarian to attend. These requests could generate long-term relationships:

“Just, we know each other better. And also, because she’d always request me to come and see them. So, 9 out of 10 visits would be me, so I know the ponies quite well as well. Their little habits and that sort of thing. I think the continuity is quite good because it’s easier to pick up changes and notice things that have changed from one visit to the next.” (Ruth, veterinarian).

“Ruth’s been coming here long enough to know that if she tells me to do something I’ll do it. So she pretty much trusts me I think, to get on with the caring without wanting to keep coming back. She knows I’ll let her know if there’s a problem.” (Lorna, owner).

Past experiences with veterinarians shaped how owners managed future relationships and the timing of veterinary involvement. Owners developed knowledge of how to respond to certain changes in their horse, and for the most part, owners dealt with issues that arose on their own. This in turn influenced the timing of any advice-seeking:

“And over the years I’ve thought to myself, and especially with Athena, “Actually, you don’t need to go rushing off for an investigation, because, at the end of the day, they just tell you to put them on box rest.” (Emma, owner).

Through experience, owners valued certain professionals to deal with certain problems. This was based upon perceptions of the professional’s area of expertise and their availability when needed. For example, some perceived a suspected foot abscess as a veterinary issue whilst others believed their farrier was best placed to resolve this problem:

“I tend to just speak to the farrier and say, “Look, I’ve got somebody who is hopping lame, wasn’t lame yesterday, can you just come out and just check for an abscess before I talk to the vet.” I tend to do that now because I find the farrier deals with an abscess probably better.” (Kathy, owner).

Not all owners wanted close relationships with their veterinarians, but all wanted a high level of expertise:

“I will get him in to do things like scoping for ulcers and different things like that. They’re not my friends. I don’t feel close. I don’t feel I could talk to them about anything. I restrict what I discuss with them.” (Jessica, owner).

Even in relationships with a veterinarian that were perceived as optimal by the owner veterinary advice was negotiated by the owner in the context of their life with their horse. The veterinarian played one part in a complex web of lay and professional advice, and through this worldview, owners constructed an understanding of what was best for their horse. Interactions with veterinarians informed owners’ ideas about their role in the management of the horse’s health.

## 4 Discussion

This study uses empirical data from the everyday experiences of owners and veterinarians and demonstrates the processes by which matters of veterinary concern are constructed. There were multiple decisions preceding the decision to call a veterinarian. The particularities of the interactions between veterinarian and owner during a consultation had consequences for the way in which veterinary expertise became integrated (or not) into horse care practices. Furthermore, the role that a veterinarian played in managing issues that arose was shaped by past interactions with veterinary professionals. Findings suggest that the impact of single interactions on the future consumption of veterinary services by owners may currently be underestimated.

Owners in this study experienced an ongoing process of assessing and reworking care strategies over time. An owner’s reasons for involving the veterinarian were temporally contingent due to the individualised and multidimensional nature of managing and monitoring the older horse. As contextual factors in the life of the human and horse are constantly interacting, co-producing and reconstructing one another in a dynamic way (18) the complex interplay between symptom recognition and actions described by Wyke et al. (32) becomes even more dynamic. Issues that became matters of veterinary concern represented a gathering of factors at a point in time (33). Analysis suggests that advice-seeking behaviours are inextricably linked to the extent to which owners perceive veterinarians to have a role in the matter of concern they have identified. In our study, single interactions appeared to have lasting effects on owners’ perceptions of veterinarians and ideas about their role in a horse’s management. These findings reflect a recent study of UK pet owners’ experiences of using veterinary services, which reported that single incidents could lead to a breakdown in trust that could prompt an owner to move veterinary practice (34). While previous work has identified the importance of the social determinants of horse health (21, 35), less attention has been paid to the role of veterinarians in shaping owner’s ideas about issues that require veterinary involvement.

Issues could become a matter of veterinary concern where a change in the horse, within the context of that individual human-horse relationship, was perceived to require veterinary expertise. In common with the complexities in human health care (36), slow change in the horse may result in difficulties in differentiating ‘normal’ from ‘abnormal’. This is likely to be individualised in nature as a result of how the horse ages and the context in which human-horse interactions take place. Zola wrote extensively about patient treatment-seeking behaviours (37). In his study of people with chronic disease he reported:

“At the time of the decision there may have been an acute episode, but this was not the first such time the symptoms had reached such a “state” but rather it was the perception of them on this occasion as interfering with the social and interpersonal relations that was the trigger or final straw.” (37).

Nevertheless, access to veterinary services when it was required was important for owners. Similar to a study of owners of dogs with chronic disease (38), owners in this study usually felt they knew when they needed to involve the veterinarian in their horse’s care. In Riley’s (39) research, farmers constructed and presented the farm, and farming practices, as embedded in multiple dimensions of past history. In this study, horse owners had a similar perspective on veterinarians and the issues that required their involvement. Issues that allowed time for reflection and consultation with other sources of advice before a veterinarian was consulted meant that a veterinarian’s visit occurred within a context filled with particular hopes, expectations, beliefs and values.

The social construction of health, disease, and the relevance of veterinary medicine to creating a ‘good’ life for a horse were found to differ between owners and veterinarians. Veterinarians made judgements about owners’ knowledge and motivation based upon decision making regarding their horse’s health. Unlike veterinarian’s intermittent and rather formulaic involvement in older horse care, owners experienced and developed their own knowledge about older horse care through their fluctuating human–horse relationship. A study involving observations of 34 routine consultations involving patients with chronic health conditions and health care professionals in Australia, found that although professionals attempted to elicit patient’s own goals for their health, there was less attention paid to patient responses that seemed unrelated to the condition itself (40). Zola (37) suggests that without attention to the factors that prompt patients to seek medical care, patients are more likely to break off from treatment plans. This was reflected in this study, where the relevance of veterinary advice to achieving good outcomes for the horse was reviewed and filtered by owners, and influenced the extent to which advice was adopted. Zola argues for the need for doctors to recognise the significance of these triggers for health care seeking even if they do not fit with the doctor’s understanding of what constitutes health and disease:

“we found that where the physician paid little attention to the specific trigger which forced or which the individual used as an excuse to seek medical aid, there was the greatest likelihood of that patient eventually breaking off treatment … being a specialist and only seeing certain kinds of problems did not exempt the physician from having to deal with this issue.” (37).

Difficulties arose for veterinarians when they perceived care was not being implemented appropriately by the owner. Veterinarians’ medically-informed perspectives about the health care needs of older horses meant that they sometimes felt conflict between serving the horse’s health and negotiating with the owner. Veterinarians in small companion animal practice report the importance of prioritising the animal through the provision of their medical care (23). However, other contextual factors such as the owner’s financial means to pay for treatment or an owner’s wishes to continue treatment in instances where a veterinarian perceives an animal to be suffering, also appear to influence veterinarians’ decision making (23). In our study, even where there was agreement between owners and veterinarians on the ‘problem’ *per se*, perspectives on the correct course of action could differ. Differences were, however, not merely due to a miscommunication but because of differences in the foundations of knowledge within which issues, and ideas about how to resolve them, were defined. Irving Zola argues that medicine’s foundations lay upon an objective truth and its claim to what is ‘real’ regarding an individual’s experience is argued to be one of the limitations of the medicalisation of life (41). This perspective may be limiting veterinarians’ acknowledgement of the complexity through which owners decide to seek their involvement in a horse’s care, and the extent to which an owner’s life with, and knowledge of, their horse shapes their views about what is best for them.

The knowledge developed by owners in their relationship with their horse is reflective of their sense of self and ability to care well for their animal. When issues were raised to necessitate veterinary involvement the perceived success of the consultation from the owner’s perspective fed back into how they managed everyday life with their horse. In common with the way in which lay knowledge is incorporated into the management of chronic disease in humans (42), horse owners’ knowledge ‘capital’ was not always mobilised or valued in the veterinary consultation. As a result, we identified evidence of owners filtering or choosing not to follow veterinary advice regarding their horse’s management. Research regarding goal-setting for people with chronic conditions suggests that the healthcare professional’s approach heavily influences what goals are valued and legitimized and the extent to which the patient plays a role in decision making (40). Veterinarian-owner interactions that result in aligned aims for the animal and trust in the veterinarian are known to be important for owners (43, 44). Relational factors such as trust and shared understanding were also found to be important in the co-production of knowledge that was more likely to be enacted by farmers (45). Therefore, the mobilisation of owners’ knowledge ‘capital’ during interactions appears to be a crucial component of successful interactions. The values that veterinarians orientate towards, and thus their behaviours towards the involvement of owners in decision making, may be a target for interventions.

Research suggest that a sense of mattering—of feeling important and feeling heard—varies with different relationship types and on an individual basis (28). In this study, single interactions could provide what an owner needed from their veterinarian. However, the specific meaning of what they needed was rooted in their relationship with their horse at that time and whether the veterinarian was able to provide for it. Where long-term relationships between veterinarians and owners were established this could create tacit knowledge about how an individual horse was ageing, the owner’s life context and their goals for the horse. Some owners spoke about valuing veterinarians in the long-term management of their horse’s health. However, even where established relationships existed much of this knowledge remained tacit and was not overtly shared in a medical record or care plan. This could prove problematic if new veterinarians unfamiliar with this knowledge attended the horse. Raising health issues of the horse to matters of veterinary concern is a multifaceted process affected by relationships. The long-term impact of single interactions with veterinarians on the uptake and future consumption of veterinary expertise may be underestimated. With increasing interest outside of the veterinary setting, mattering as a predictor of outcomes may be an important concept for the veterinary profession to consider (28). Veterinary work within a complex network of multispecies relationships, involving animal, owner, profession and society, also offers a novel setting for future enquiry in the particularities of mattering.

The limitations of this study include the collection of data from a relatively small subset of the equestrian and veterinary communities. Interview participants were selected based on certain inclusion criteria and the online advertisement for participation may have also impacted on who had access to participate in the study. In addition, there was a lack of male owners recruited, and although reflective of the demographics of the wider horse-owning community (46), it may have been useful to strategically sample from this group. Veterinarians in the study were sampled based on their relationship with a particular owner who had volunteered. Recruitment of veterinarians was difficult (either due to a failure to respond to invitation, or declining due to a lack of time) and therefore the sample size was relatively small. However, as the methodological approach was adopted to generate in-depth understanding rather than a generalisable ‘truth’ the sample obtained was satisfactory for this purpose.

## 5 Conclusion

Matters of veterinary concern were the product of the constant interaction, co-production and reconstruction of contextual factors in the life of the owner and their older horse. There is a distinction between matters of concern and the point at which they are deemed to be a matter of veterinary concern. Veterinarians’ and owners’ divergent experiential contexts come together where consultations take place and their interplay affects the extent to which veterinary expertise becomes integrated (or not) into horse care practices.

Owners’ sense of mattering was affected by interactions with veterinarians, and this affected the nature and timing of their advice-seeking behaviours. Findings indicate that developing, and maintaining, a sense of mattering for both owners and veterinarians will be important if different types of expertise in relation to older horse health are to be brought together as an animal ages. The long-term impact of single interactions on the future consumption of veterinary services requires further investigation.

## Data availability statement

The raw data supporting the conclusions of this article will be made available by the authors, without undue reservation.

## Ethics statement

The studies involving humans were approved by University of Liverpool’s Veterinary Research Ethics Committee. The studies were conducted in accordance with the local legislation and institutional requirements. The participants provided their written informed consent to participate in this study.

## Author contributions

RS: Conceptualization, Data curation, Formal analysis, Investigation, Methodology, Project administration, Resources, Software, Validation, Visualization, Writing – original draft, Writing – review & editing. GP: Writing – review & editing, Supervision, Funding acquisition, Conceptualization. CM: Writing – review & editing, Supervision, Funding acquisition, Conceptualization. JI: Writing – review & editing, Supervision, Funding acquisition, Conceptualization. EP: Writing – review & editing, Validation, Supervision, Methodology, Funding acquisition, Formal analysis, Conceptualization.
