# Supplementary material for: Becoming a matter of veterinary concern
Source: Front Vet Sci. 2024 May 30;11:1355996. doi: 10.3389/fvets.2024.1355996 (PMC11169876; doi:10.3389/fvets.2024.1355996)
Supplement: Supplementary file 1 [file Data_Sheet_1.docx]

Supplementary Material

Becoming a matter of veterinary concern

Rebecca Smith*, Gina Pinchbeck, Catherine McGowan, Joanne Ireland, Elizabeth Perkins

*** Correspondence:** [**rebecca.smith2@liverpool.ac.uk**](mailto:rebecca.smith2@liverpool.ac.uk)

# Supplementary Data 1

**Participant consent form owner: Part 1**

**Title of Research Project: Understanding and improving care of the older horse**

Researchers: Dr Gina Pinchbeck, Rebecca Smith, Professor Elizabeth Perkins, Professor Cathy McGowan, Dr Jo Ireland

Please initial box

1. I confirm that I have read (or had it read to me) and have understood the information sheet dated April 2020 for the above study.
2. I understand that taking part in the study involves an interview which will be audio-recorded, and understand that the audio recording will be transcribed as part of the study.
3. I understand that my participation is voluntary and that I am free to decline to answer any particular question, or to stop taking part in the interview at any time without giving any reason.
4. I understand that I can ask for access to the data generated from the interview and I can request the destruction of that data if I wish, up to two weeks following my interview.
5. I understand that the personal and anonymised data I provide, including signed consent forms, audio recordings and transcripts, will be held securely and in line with data protection requirements at the University of Liverpool.
6. I agree for the data collected from me to be shared within the research team and used in future research, if reviewed and approved by a research ethics committee. Data will be stored for up to 10 years by the University of Liverpool.
7. I agree to be contacted at a later date:
8. If the research team wishes to clarify details from the interview recording.
9. For the purposes of invitation to take part in future studies of a similar nature. I understand that I am under no obligation to take part in any future studies.
10. I agree to take part in the above study.

Participant name Date Signature

Name of person taking consent Date Signature

**Participant consent form owner: Part 2**

**Title of Research Project: Understanding and improving care of the older horse**

Researchers: Dr Gina Pinchbeck, Rebecca Smith, Professor Elizabeth Perkins, Professor Cathy McGowan, Dr Jo Ireland

1. I agree for the research team to request the veterinary clinical records for my horse(s) from my veterinary practice(s). *Detail below.*

| Horse(s) name (stable and passport name if different) | Name of veterinary surgeon(s) | Name and address of veterinary practice(s) |
| --- | --- | --- |
|  |  |  |

2. I agree for the research team to contact my veterinary surgeon(s) and/or retirement yard owner(s) if applicable, to request their participation in the study.

| Name and contact details for veterinary surgeon(s) | Name and contact details for retirement yard owner |
| --- | --- |
|  |  |

Participant name Date Signature

Name of person taking consent Date Signature

**Participant consent form vet**

**Title of Research Project: Understanding and improving care of the older horse**

Researchers: Dr Gina Pinchbeck, Rebecca Smith, Professor Elizabeth Perkins, Professor Cathy McGowan, Dr Jo Ireland

Please initial box

1. I confirm that I have read (or had it read to me) and understood the information sheet dated April 2020 for the above study.
2. I understand that taking part in the study involves an interview which will be audio-recorded, and understand that the audio recording will be transcribed as part of the study.
3. I understand that my participation is voluntary and that I am free to decline to answer any particular question, or to stop taking part in the interview at any time without giving any reason.
4. I understand that I can ask for access to the data generated from the interview and I can request the destruction of that data if I wish, up to two weeks following my interview.
5. I understand that the personal and anonymised data I provide, including signed consent forms, audio recordings and transcripts, will be held securely and in line with data protection requirements at the University of Liverpool.
6. I agree for the data collected from me to be shared within the research team and used in future research, if reviewed and approved by a research ethics committee. Data will be stored for up to 10 years by the University of Liverpool.

I agree to be contacted at a later date:

1. If the research team wishes to clarify details from the interview recording.
2. For the purposes of invitation to take part in future studies of a similar nature. I am under no obligation to take part in any future studies.
3. I agree to take part in the above study.

Participant name Date Signature

Name of person taking consent Date Signature

# Supplementary Data 2

**Interview topic guide: Horse owners**

*Introduction to interview*

*In this study we are interested to know more about your experiences and beliefs, and how you have come to manage your previous or current horses as they have got older. We are keen to understand what you believe to be important when caring for an older horse.*

**Scene setting/experience**

- Can you tell me about your experience of caring for different horses?

**Management & care**

- Could you describe any special accommodations you have made for your horse as (s)he has aged? (Prompts: What made you decide to change your management practice?)
- How have you decided on the healthcare your horse(s) receives?
- Did you previously plan how you would manage your horse in its older years?

**Horse-human relationship**

- How would you describe your relationship with your horse(s)? (Prompts: Does this differ between each horse? How has this relationship varied over time?)
- Can you describe what you think ‘old age’ is for a horse? (Prompts: What do you think is important to an older horse? How do you think a horse is expected to live in old age?)

**Quality of life**

- What do you think quality of life means for a horse? (Prompts: What factors do you think are important for your horses’ quality of life? In what ways may these factors change over time?)

**Owner-vet relationship**

- How would you decide if you needed to act upon something different you had noticed in your horse? [If owner has experience with horses of different ages - How would you act with an older horse compared to a younger horse?]
- If you had a concern about your horse’s health or behaviour where would you go to for advice?
- What kind of relationship do you have with your vet(s)? (Prompts: In what instances would you value your vets’ opinion? How have any previous experiences affected how you interact with your vet?)
- As your horse has aged, has this changed the relationship you have with your vet?

**Euthanasia**

- Have you previously had to make the decision to have a horse put to sleep?
- (*If so*) Can you explain how you came to make the decision to put your horse to sleep? (Prompts: Had you discussed this with anyone? What do you think are important factors to consider when making the decision?)
- (*If not*) What are your views on euthanasia in horses? (Prompts: What do you think are important factors to consider when deciding whether euthanasia is appropriate for a horse? Do you think that you would seek advice when making this decision? If so, where would you seek advice?)
- What do you think is meant by providing a ‘good death’ for a horse?

**Closing questions**

- From your experience, what advice would you give to someone who is caring for an older horse?
- Is there anything that you might not have thought about before that has occurred to you during this interview?
- Is there anything else you would like to add?
- Is there anything you would like to ask me?

**Interview topic guide: Veterinary surgeon**

*Introduction to interview*

*We know that as a horse gets older they can experience changes in management and in veterinary intervention. In this study we are interested to know more about your approaches and experiences with older horses, to get an idea of what is important to you when caring for these patients.*

*Horse x and owner x to be substituted with relevant participant names.*

**Management experiences and vet-owner relationship**

- Could you describe your involvement in the care and management of *horse x*?
- Does *horse x* have any health or wellbeing issues of concern?
- Do you think that you and *owner x* have the same views on how to manage these issues?
- How do you think *owner x* sees your role in the care of *horse x*? (Prompts: Do you think your advice is valued?)

**Horse-human relationship and healthcare**

- Do you think that older horses have different care needs compared to younger animals? (Prompts: Could you describe any barriers to older horses receiving appropriate care?)
- How might your priorities and attitudes towards care change when treating an older horse? (Prompts: Would you give certain factors more weight when making decisions?)
- What do you think are the main welfare concerns surrounding older horses? (Prompts: Can you hypothesise as to why these issues may occur?)

**Quality of life and euthanasia**

- How would you assess quality of life in an older horse? (Prompts: Have your approaches to this changed over time?)
- How do you think *owner x* assesses quality of life in *horse x*?
- What do you think the vet’s role is surrounding euthanasia decisions in an older horse? (Prompts: How might the vets’ expertise be better utilised?)
- What do you think is meant by providing a ‘good death’ for a horse?

**Closing questions**

- Is there anything that you might not have thought about before that has occurred to you during this interview?
- Is there anything else you would like to add?
- Is there anything you would like to ask me?
